# Engagement in computerized cognitive training instructions by older people. A within-subject design to evaluate comprehension and acceptability of serious games instructions

**DOI:** 10.3389/fragi.2025.1297704

**Published:** 2025-02-03

**Authors:** Christelle Nahas, Marc Gandit, Emmanuel Monfort

**Affiliations:** ^1^ Translational Innovation in Medicine and Complexity (TIMC, UMR 5525), Université Grenoble Alpes, CNRS, Grenoble, France; ^2^ Laboratoire InterUniversitaire de Psychologie (LIP/PC2S, EA4145), Université Grenoble Alpes, Grenoble, France

**Keywords:** computerized cognitive training, visual cues, instructional comprehension, software acceptance, engagement, older adults, within-subject design

## Abstract

This article emphasizes the advantages of using a within-subject experimental design to assess the impact of salient visual cues on the comprehension and acceptability of computerized cognitive training (CCT) instructions among older adults. The study would involve participants aged 65 and above, who will engage in an online experiment presenting two sets of instructions for serious games: one with salient visual cues and one without. This within-subject design eliminates the need for random assignment, improves internal consistency, and enhances statistical power. Participants serve as their own controls, providing a more robust comparison of how visual cues affect instruction comprehension and software acceptance. The primary objective is to identify indicators of acceptability for CCT serious games and to evaluate how well participants comprehend the instructions, influencing their intention to use the software. The hypothesis suggests that salient visual cues will improve instruction comprehension and foster greater software acceptability. By focusing on this design method, the study aims to enhance the engagement of older adults in cognitive training programs, reducing dropout rates. This research offers valuable insights into methodological strengths that can be applied in future studies to improve the usability and acceptance of CCT tools for older adults without cognitive impairments.

## 1 Introduction

In recent years, computerized cognitive training (CCT) has become a popular tool to cope with declining cognitive functions in a variety of contexts. One particularly promising area of application is in rehabilitation, where CCT has shown potential for improving cognitive abilities in older adults with cognitive impairments (Hill et al., 2017; Lampit et al., 2014; Saragih et al., 2022).

While there is some evidence indicating cognitive improvement for older adults with cognitive impairments using CCT (Hill et al., 2017; Saragih et al., 2022; Hu et al., 2019; Kelly et al., 2014), there are still difficulties in measuring the transferability of this progress to daily life activities (Saragih et al., 2022; Coyle et al., 2015; Lai et al., 2021; Webb et al., 2018). Daily life activities are essential for human functioning and affect autonomy when they cannot be realized. The difficulties related to measuring this transferability are linked to several factors that could be methodologically related. For example, the lack of standardized outcome measures across studies, the variability in training programs, the limited duration of training, and the individual differences in cognitive abilities among participants are all factors that may contribute to difficulties in measuring the transferability of cognitive gains (Hill et al., 2017; Lampit et al., 2014; Webb et al., 2018; Birney et al., 2015; Sigmundsdottir et al., 2016).

Adhering to cognitive training programs can be challenging due to various factors, including lack of support, resources, and coping skills (Deao, 2017; Lequerica et al., 2010). According to Lequerica and Kortte (Lequerica et al., 2010), engaging in cognitive rehabilitation requires three essential elements that lead to behavioral intention or willingness to comply with treatment: (Hill et al., 2017): the perception of rehabilitation needs, (Lampit et al., 2014), the outcome expectancy for the treatment, and (Saragih et al., 2022) the perception of self-efficacy. Similarly, for older adults, adherence to computerized cognitive training is influenced by factors like previous computer use, cognitive capacity, and social support (Turunen et al., 2019). Thus, interventions that are too intellectually demanding increase the risk of dropout, suggesting that facilitating computer use and providing extra support to those with cognitive difficulties could improve adherence. Being socially supported, having easy access to the programs and being offered adaptations according to the difficulties encountered can encourage efficient use of cognitive training programs. Additionally, the perceptions of participants regarding the benefits and purpose of computerized cognitive training, including their understanding and acknowledgment of their own deficits, were identified as crucial factors that influence the use of CCT (Srisuwan et al., 2020).

### 1.1 The necessity of factoring in engagement when designing computer-based support environments for older people

Engagement is characterized by effortful participation, indicating the deployment of energy by an individual to achieve a goal (Filsecker and Kerres, 2014). It can continue through persistent re-engagement (Garris et al., 2002), which translates into continued involvement in the task, engagement in the face of challenges, and sustained effort. In the context of using digital tools, engagement is defined as the energy investment directed by an individual towards a particular stimulus or task (Nahum-Shani et al., 2022). An individual’s level of engagement is not constant and can vary depending on the nature of the activities performed (Reina-Tamayo et al., 2017).

Engagement is a multidimensional concept that includes three main dimensions: physical (or behavioral) engagement, affective engagement, and cognitive engagement, each contributing uniquely to the overall dynamics of engagement (Kelders et al., 2020; Perski et al., 2017; Yardley et al., 2016). In digital health interventions, engagement characterizes the ability to follow prescribed treatments (physical engagement), feel emotional wellbeing (affective engagement), and fully understand the importance and functioning of treatments (cognitive engagement) to achieve an optimal level of engagement (King et al., 2014).

Acceptance, on the other hand, encompasses broader psychological factors influencing a user’s decision to adopt technology, both before and after initial use, which includes their level of engagement (Alexandre et al., 2018; Mascret et al., 2020). While engagement is a component of acceptance, it is distinct in its focus on the active and ongoing participation in specific tasks. For instance, in the context of a CCT tool, acceptance heavily relies on user familiarity and ease of use; a lack of understanding of its functionality can hinder acceptance (Shi et al., 2021; Torous et al., 2018). Key components of engagement, such as perceived need, expected outcomes, and self-efficacy in using the technology, serve as critical predictors of its overall acceptance (Torous et al., 2018; Kabir et al., 2016; Nadal et al., 2020; Venkatesh et al., 2012; O’Brien et al., 2008; Davis et al., 1986).

Older adults’ engagement can impact their cognitive performance. Age-related differences in performance are often due to decreased cognitive control (Braver et al., 2002). Older adults struggle more with maintaining task goals and updating them based on changing situations (Braver et al., 2008), which can be explained by the influence of cognitive load on the regulation of this engagement. Moreover, declining motivation to engage in cognitively demanding activities is common with aging (Depping et al., 2012). According to Sweller and Chandler (1991) learning efficiency can be improved by reducing extrinsic cognitive load (unrelated mental effort) and maximizing germane cognitive load (effort beneficial to learning). Excessive cognitive load occurs when learning material exceeds the learner’s capabilities, resulting in poor learning outcomes (Sweller and Chandler, 1991).

Studies on engagement in rehabilitation highlight the significance of an individual’s perception of need, expected outcomes, and self-efficacy in maintaining engagement (Lequerica et al., 2010). Choi and Twamley (Choi et al., 2013) examined methods aimed at improving treatment engagement and self-efficacy in their review of Cognitive Rehabilitation Therapies for Older adults with Alzheimer’s disease. They identified barriers to cognitive rehabilitation engagement, such as low self-efficacy and a tendency to expect failure (outcome expectancy), which significantly influence goal-directed and task-centered behavior. Another barrier is the individual’s understanding of the purpose and value of specific training tasks or treatment programs (perceived need/usefulness).

Since CCT involves using technology, engagement is influenced by usability, which refers to factors such as task completion time, user satisfaction, and ease of learning. Poor usability can lead to low user engagement and adherence by affecting the perception of self-efficacy, as users may find the technology challenging to use (Torous et al., 2018). It can also impact the perception of need, as older users may not understand the technology’s utility due to poor usability. For example, Guner and Acarturk (Guner and Acarturk, 2020) found that older adults placed more importance on the perceived usefulness of technology and its ease of use, which positively influenced their attitudes and acceptance. Additionally, poor usability can affect the perception of outcome expectancy by decreasing user trust if they struggle to use the technology and do not fully grasp its potential benefits (O’Brien et al., 2008). This is especially true for older adults. When technology is difficult to use or does not align with their preferences and abilities, it can significantly influence their sense of self-efficacy in using the technology (Vette et al., 2019).

Overall, the usability of CCT tools determines engagement, especially for older adults who value ease of use and perceived usefulness. Poor usability can lower self-efficacy and acceptance, reducing engagement. User-friendly design and clear communication of benefits are essential for maintaining engagement, particularly in rehabilitation settings. Thus, CCT tools should be easy to use and tailored to user preferences to enhance engagement.

### 1.2 Improving CCT instructions to increase engagement through the use of saliency

Cognitive aging results in a decline in decision-making abilities (Fechner et al., 2019; Hartshorne et al., 2015), attention (Geerligs et al., 2014; Lezak et al., 2004), mental flexibility, and inhibitory processes (Collette and Salmon, 2014; Turner et al., 2012), which are essential for understanding instructions. Specifically, aging affects flexibility, the ability to form, change, and update representations in working memory (Fechner et al., 2019), thereby reducing executive functioning across a wide range of cognitive tasks such as planning, problem-solving, and multitasking (Glisky et al., 2007).

High cognitive load makes complex tasks more difficult for older adults who may also experience difficulties maintaining concentration or filtering out distractions during demanding tasks (Hasher et al., 1988; Salthouse et al., 1996). This is also observed in studies by Wynn, Amer, et al. (2020) which indicate that older adults tend to be more easily distracted by salient external cues, such as bright or moving objects, even when asked to ignore them (Wynn et al., 2020). This tendency is attributed to a decline in controlled inhibition, leading to difficulty suppressing rapid eye movements. When trying to avert their gaze from a sudden peripheral cue, older adults make more errors, indicating a reduced ability to control their eye movements. In addition, age-related changes in visual behavior, resulting from diminished cognitive control and inhibition, can contribute to the memory deficits observed in older adults. Prolonged exposure to distracting stimuli can saturate memory representations, thereby increasing the tendency to remember information consistent with pre-existing schemas rather than based on factual accuracy (Amer et al., 2020).

Instructions play a major role in understanding and performing a task, and may therefore be difficult for older people to understand. In particular, given that older adults may have reduced attention capacity, instruction design should focus on directing attention to essential material while minimizing distractions. Strategies include making important stimuli salient and emphasizing the value and location of key information sources (Sharit et al., 2020). Instructional design affects information processing, therefore, holds a significant influence over comprehension (Ganier, 2004; Ganier, 2013a; Ganier, 2013b; Ganier et al., 2000; Sohlberg et al., 2011). Comprehension is an important pilar for effective usability (Kabir et al., 2016) as well as acceptability (perceived ease of use, self-efficacy, outcome expectancy) (Lequerica et al., 2010; Alexandre et al., 2018; Nadal et al., 2020; Venkatesh et al., 2012). Focusing on instructional design, could address cognitive and perceptual barriers associated to technology use by older adults such as: capacity of using technology (e.g., navigation, learning); information processing; memory; language (e.g., choice of wording) and visual needs (e.g., font size, colors) (Farage et al., 2012; Morey et al., 2019; Nurgalieva et al., 2019).

Universal design, as proposed by Gassmann and Reepmeyer (2011), encompasses seven key principles: Equity, Flexibility, Simplicity, Perceptibility, Error Recovery, Low Effort, and Accessibility. These principles address various aspects of design to create inclusive and user-friendly solutions. Equity emphasizes the importance of providing equal utility without stigmatization, catering to the needs of all individuals (Gassmann et al., 2011). Flexibility ensures adaptability and the availability of choices to accommodate diverse preferences. Simplicity aims to enhance usability by simplifying the user experience. Perceptibility guarantees effective communication of essential information. Error recovery seeks to minimize mistakes and enable easy rectification. Low effort focuses on crafting a comfortable user experience that minimizes strain. Finally, accessibility ensures that the design accounts for variations in physical abilities. Moreover, the main elements to consider when presenting information to older adults are simplicity, intuitive logic (consistency within procedures), moderate pace, and a minimum of non-relevant information (Farage et al., 2012). More specifically, since difficulties related to information processing and memory but also perceptive skills related to visual abilities are likely to affect the understanding of instructions (Ganier, 2013a; Farage et al., 2012; Azevedo et al., 2022), identifying what can be a barrier to these aspects and addressing them could facilitate the process of understanding instructions in CCT use.

Among the information that facilitates attentional processing, location-based cues generally improve performance in terms of reaction time (McLaughlin et al., 2010). They facilitate performance in both young and older adults, regardless of the presence of the target and the number of distractors. This suggests that salient visual cues help by automatically focusing attention, in contrast to more complex conjunction search conditions where a different pattern of results was observed. In more difficult search conditions, older adults benefited from cues by developing effective search strategies, especially when the number of distractors was reduced. Younger participants did not consistently benefit from salient visual cues in complex search conditions, suggesting either variance in the ability to use the cues or inherent efficiency in their search strategies that did not depend on external cues.

Although the capture of attention by salient and irrelevant cues can be adaptive, for example, by signaling potential danger or reward, it can nevertheless divert attention from relevant tasks, impairing memory performance in daily activities. Moreover, older adults show increased sensitivity to cues based on their expectations and prior knowledge, which can lead them to focus their attention on anticipated locations even in the absence of the searched-for object. This preference for internal cues can result in longer search times and reduced memory accuracy (Wynn et al., 2020; Chan et al., 2018; Liu et al., 2018).

### 1.3 Our contribution

The notion that instructions can affect comprehension and that salient visual cues direct attention to specific elements led us to study how the presence or absence of such cues influences the acceptability and comprehension of instructions for CCT exercises. We also aim to understand how intrapsychic factors, such as perceived self-efficacy and anxiety, influence these elements as well. This resulted in the design of two distinct types of instruction models developed for CCT exercises. These proposed designs both emphasize goal prioritization and the sequential arrangement of information, as recommended by literature (Ganier, 2013a; Ganier, 2013b; Sohlberg et al., 2011; Sohlberg et al., 2005). The sole distinction between the models lies in the inclusion or exclusion of prominent visual cues. To the best of our understanding, no previous research has explored the instructional format linked to the usage of a CCT program.

To our knowledge, no previous research has directly investigated the methodological aspects of instructional formats specifically linked to the use of CCT programs. Consequently, the purpose of this article is to introduce a method aimed at achieving two objectives. First, we seek to investigate the impact of salient visual cues on instructional comprehension for people aged over 65, without any known diagnosis for cognitive impairment. Second, we aim to examine the influence of these cues on software acceptance. To accomplish this, we will employ an online survey platform and present two types of instructions: one with visual cues and another without visual cues. This methodological approach allows direct comparison of the effects of visual cues in a controlled, within-subject design. Our primary goal is to identify acceptance indicators, including perceived need, outcome expectations, self-efficacy, and intention to use a CCT software. Additionally, we aim to assess if differences in comprehension play a significant role in shaping acceptance behavior. Through this study we hope to gain a deeper understanding of how these factors influence the intention to use this cognitive training tool by people aged over 65. By doing so, we aim to enhance engagement in cognitive training and reduce dropout rates for this target population.

Our hypothesis states that including salient visual cues, in the form of analogical help indicators, will enhance comprehension and acceptance of the instructions for using CCT software among individuals aged over 65 without cognitive impairments. We anticipate that instructions featuring salient cues will result in better understanding and acceptance compared to those without such cues. Additionally, we expect that participants’ self-efficacy and their levels of technology-related anxiety will influence their acceptance and understanding of the instructions. By employing this method, we aim to provide a clearer understanding of how these factors interact to influence user engagement and acceptance. Specifically, we predict that higher self-efficacy—whether in their cognitive abilities or in using technology—will correlate with greater acceptance and intention to use the software. Conversely, higher technology anxiety is expected to be associated with lower acceptance and intention to use the software. By exploring these relationships, our study aims to uncover the significance of self-efficacy and technology anxiety in shaping users’ responses to instructional cues within the context of CCT software. This methodological exploration aims to uncover how these variables interact within the context of instructional design for CCT software, offering valuable insights for future research and application.

## 2 Materials and equipment

This study adopts an online within-subject design, where each participant serves as their own control and experiences multiple conditions. This approach eliminates the need for random assignment since every participant experiences all conditions, in a counter-balanced way, allowing for direct comparisons within each individual. This strengthens internal validity, making the findings more robust and statistically powerful in detecting significant effects and reduces the requirement for an exceptionally large sample size (Charness et al., 2012; Lindner et al., 2022). This methodology is particularly interesting for other researchers because it is designed to be conducted online, which allows for a broader reach of participants. Additionally, it provides a valuable framework for designers to test different instructional methods and gather critical information on their acceptance before incorporating them into their designs.

### 2.1 Participants

Participants will be adults aged 65 years or older, without any diagnosed neurocognitive disorders. They must have access to a touchscreen tablet or a computer with internet connectivity, and phone use for questionnaire completion will be discouraged to ensure proper presentation of the instructions. Non-native French speakers and individuals with uncorrected visual or hearing impairments will be excluded from the study.

To determine the appropriate sample size for our study, we conducted a power analysis using G*Power 3.1.9.7 software, focusing on an ANOVA with repeated measures within-between interaction. We set the effect size (f) to 0.20, the alpha error probability to 0.05, and aimed for a power of 0.80. Our study design included two groups, with each subject measured 6 times, assuming a correlation of 0.3 among repeated measures and a nonsphericity correction of 1. The analysis indicated that a total sample size of 40 subjects would be sufficient, yielding a noncentrality parameter (λ) of 13.71, a critical F value of 2.26, with numerator and denominator degrees of freedom at 5 and 190, respectively. This configuration results in an actual power of approximately 0.82, ensuring that our study is well-powered to detect the specified effect size with acceptable Type I and Type II error rates.

### 2.2 Materials

#### 2.2.1 Instructional design

The instructions designed for this experiment were inspired by the COVIRTUA Cognition-Intersession^©^, a CCT software used by cognitive rehabilitation professionals (Nahas et al., 2023) and still in the developmental phase. This tool operates on two separate screens: a patient screen, consisting of a touch tablet equipped with a pen, and a professional screen, which is a personal computer. COVIRTUA Cognition-Intersession^©^ is designed to train cognitive functions and includes exercises targeting specific cognitive abilities such as attention, memory, and language. Additionally, it incorporates exercises that simulate everyday activities using virtual reality technology.

In the COVIRTUA Cognition-Intersession^©^ system, the user, who is typically a patient, engages in exercises that are programmed by a rehabilitation professional. The user interacts with a simplified screen that only displays the necessary elements for performing the exercises. On the other hand, the professional, which can be an occupational therapist, a speech therapist, a psychomotor therapist, or a neuropsychologist, selects the activities in advance, determines their difficulty levels, and reviews the results on their own screen. Additionally, professionals have the ability to manage user files, configure settings, and program exercises. This system enables personalized adaptation of activities based on individual needs and preferences. For a more comprehensive description, please refer to: https://www.covirtua.com/solution. Specifically, we will utilize exercises available on the CCT software to conduct our online experiments. The instructional designs chosen for both studies are prototypes that have not yet been implemented in the software.

In this study, six CCT serious games were selected, comprising three analytical exercises and three functional exercises. The analytical exercises, which include *Barrage* (Cancellation), *Le Bon Groupe* (The Right Group), and *Memory*, are designed to mobilize fundamental cognitive skills through decontextualized tasks. On the other hand, the functional exercises, which consist of *La Liste de Course* (The Shopping List), *Les Courses* (Shopping at the Supermarket), and *Le GPS* (Following a GPS Route), simulate everyday activities and engage multiple cognitive capacities simultaneously. Detailed descriptions of these exercises, including their French titles, can be found in the Supplementary Material.

**FIGURE 1:**
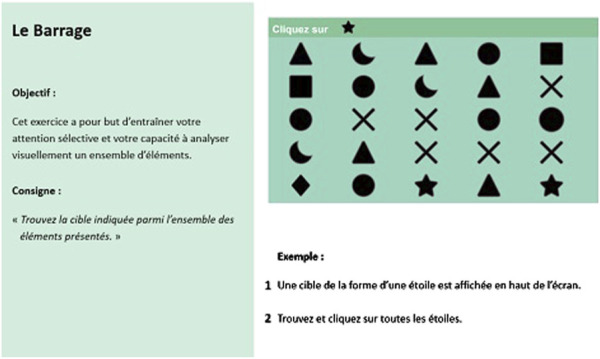
Example of analytical exercise “Le Barrage” without any salient visual cues (Modality A).

**FIGURE 2:**
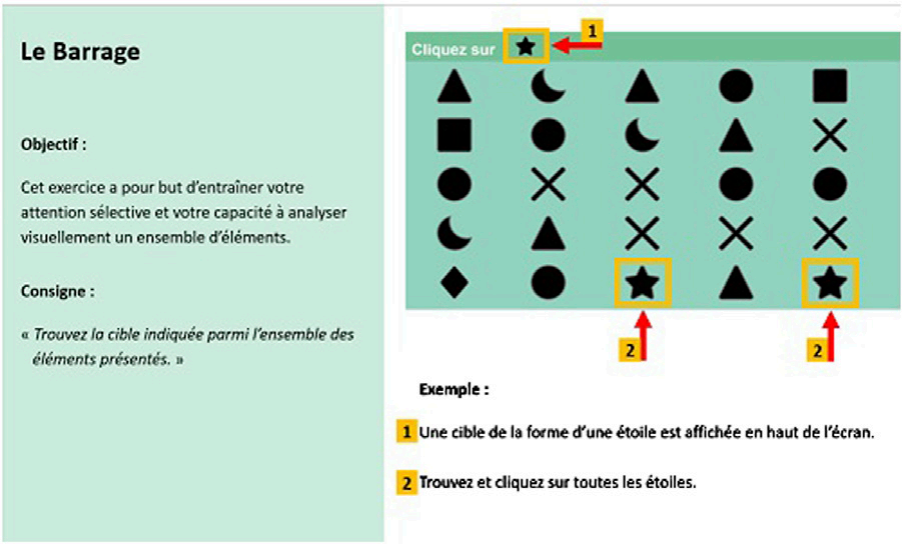
Example of analytical exercise “Le Barrage” with salient visual cues (Modality B).

Each exercise’s instructions are divided into two main parts: the objective of the exercise and the description of the procedure to perform the exercise. These instructions will be presented in two distinct modalities to examine the impact of visual cues on task comprehension and execution. Modality A involves presenting the information without salient visual cues, as illustrated in Figure 1. In contrast, Modality B includes the presentation of information with salient visual cues such as arrows, colors, and shapes, as shown in Figure 2. Both instructional elements (objective and procedure) will be combined and displayed in a single image to facilitate a clear comparison between the two modalities.

The instructional material was designed by the first author, a PhD student without professional design training. To minimize bias, the design incorporated several recommendations from the literature.

- Multimedia Usage: Instructions combine pictures and text or pictures and audio to facilitate information processing (Ganier, 2004; Ganier, 2013a; Ganier, 2013b; Chowdhury et al., 2021; Mayer and Moreno, 2005).
- Cognitive Load Reduction: Information is minimized and broken down into smaller steps to reduce cognitive load (Sohlberg et al., 2011; Farage et al., 2012; Azevedo et al., 2022; Sohlberg et al., 2005; Chowdhury et al., 2021; Mitzner et al., 2019).
- Highlighting Relevant Information: Relevant information is emphasized, while irrelevant and distracting information is minimized (Morey et al., 2019).
- Simple Text and Images: Simple text and images are used to avoid misinterpretation (Farage et al., 2012).

These strategies collectively enhance the clarity, accessibility, and reliability of the instructions, ensuring the material is fair and effective for a diverse audience, so that we can effectively focus on studying the influence of the salient visual cues.

Both Modality A and Modality B adhere to these design principles. The content, including the objectives and procedures for each exercise, was defined by the COVIRTUA Cognition software. The only difference between the modalities is the presence of visual cues in Modality B. Visual cues such as arrows, colors, and shapes were chosen based on the notion that older adults are more susceptible to attention capture by salient visual cues compared to younger adults. This choice is further supported by additional design recommendations for older adults found in the literature.

- Use of Colors, Icons, and Graphics: Prioritizing visual elements over text for displaying information (Wynn et al., 2020; Carmien et al., 2014; Davis and Ohman, 2016).
- Visibility of Frequent or Important Actions: Making key actions readily visible and accessible (Farage et al., 2012).
- Pop-up Hints and Help Bubbles: Considering the use of these features for new tasks (Morey et al., 2019).

By structuring the instructions and exercises in this manner, the study aims to comprehensively evaluate how visual cues affect participants’ cognitive performance and their engagement with the serious games. This approach will provide valuable insights into the effectiveness of different instructional designs in cognitive training for older adults. For a detailed review of all instructional material and the associated English translations, please refer to the Supplementary Material.

#### 2.2.2 Questionnaires

The study will be conducted entirely online using the LimeSurvey online survey platform, compliance with European data protection regulations. The online procedure utilized for this research, is constructed in accordance with the CHERRIES criteria (Eysenbach, 2004), which are recommended for improving the quality of research conducted through online questionnaires. Data collection is divided into four main parts.

1- Sociodemographic questionnaire required to fulfill the scientific objectives of this project, such as age, gender, relationship status and educational level
2- Questions for control purposes:
    a. An assessment of the subjective cognitive complaint, to measure cognitive self-efficacy, using the subjective cognitive complaint questionnaire (SCCQ) (Thomas-anterion et al., 2004);
    b. An evaluation of the general feeling of self-efficacy for the use of a technology, using the self-efficacy scale developed by (Compeau et al., 1995) and translated into French by Faurie et al. (2007).
    c. An evaluation of technology anxiety, using the four items of the Computer rating scale (Heinssen et al., 1987), translated into French by (Loorbach et al., 2015);
3- The presentation of the instructions for six CTT serious games (three analytical and three functional exercises), according to two modalities, presented randomly (modality A or B- check Figures 1, 2 for an example);
    a. Each presented instruction is followed by an evaluation of the acceptance and intention to use for a program of six CCT exercises of the COVIRTUA cognition software, coupled with questions related to the perceived comprehension of the instructions (Table 1);
4- An evaluation of instruction retention through exercise-specific questions;

**TABLE 1:** List of questions asked after the presentation of each instruction for the six CCT serious games.

Question	French version	English version	Indicators	References it was adapted from
1	La disposition des informations dans l’ensemble de la page contribue à maintenir mon attention	The way the information is arranged on the pages helped keep my attention	Perceived comprehension	RIMMS; Loorbach et al. (2015)
2	L’organisation des informations partagées me rend confiant(e) dans ma capacité à comprendre comment réaliser cet exercice	The good organization of the content helped me be confident that I would learn this material	Perceived comprehension	RIMMS; Loorbach et al., 2015
3	Je trouve cet exercice d’entraînement cognitif utile	I find this cognitive training exercise useful	Acceptability (Perceived usefulness)	Hayotte et al. (2020)
4	Je trouve qu’apprendre à réaliser cet exercice est facile pour moi	I find this exercise easy to learn	Acceptability (Self-efficacy)	Hayotte et al. (2020)
5	Je pense que cet exercice va me permettre de développer mes compétences	I think this exercise will help me develop my skills	Acceptability (Outcome expectancy)	Hayotte et al. (2020)
6	Je trouve que les consignes sont faciles à comprendre	I find the instructions easy to understand	Acceptability (Effort expectancy/Self-efficacy)	Castilla et al. (2018)

Additionally, an awareness message will be displayed to participants at the end of the questionnaire, that also offers the option to contact the research team for further guidance if desired.

The SCCQ questionnaire consists of 10 items. A score above three indicates a potential need for cognitive assessment or supervision, while a score below three suggests normal cognitive functioning. It is important to note that the SCCQ is not a diagnostic tool, and participants’ scores will not be disclosed. The reason for using the subjective cognitive complaint questions by (Thomas-anterion et al., 2004), is to ensure that the comprehension of the instructions or the acceptance indicators will not be affected by the possibility of any biases related to possible cognitive impairment. This is done because our inclusion criteria are only based on the absence of any cognitive impairment diagnosis, we will not collect any official medical document to support that, the participant will simply be informed in the information notice and will be asked the question explicitly at the beginning of the questionnaire.

The questions following the presented instructions were carefully crafted to assess participants’ perceived comprehension and acceptability of the CCT serious games. We used questions derived from the Reduced Instructional Materials Motivation Survey (RIMMS) to gauge instructional comprehension (Loorbach et al., 2015). These questions were originally in English, and were then translated by a bilingual member of the research team. Additionally, we included questions related to acceptability and intention to use the software, measuring factors like perceived need (usefulness), outcome expectations, and self-efficacy adapted from the French validation of the eHealth acceptability scale (Hayotte et al., 2020) as well as inspired by elements of the technology acceptance model (Davis et al., 1986) found in a case study by Castilla et al. (2018) on a digital literacy method for the elderly (Also translated and adapted from English to French). We did not include all the questions from each above-mentioned references (Loorbach et al., 2015; Hayotte et al., 2020; Castilla et al., 2018) in order to reduce cognitive load by minimizing the number of questions asked as much as possible. This resulted in the selection, and adaptation of six questions including at least one of the targeted indicators following each presented instruction (six serious game instructions in total; Table 1).

Finally, towards the end of the online evaluation, inquiries will be carried out regarding the retention of information presented in the instructions. This serves as a control measure to assess the participants’ actual comprehension of the instructions. Asking questions about the material is a precise way to measure retention because it directly tests the participants’ recall and understanding of key concepts. It ensures that the information was not only read but also understood and remembered, providing a clear indicator of instructional effectiveness. The questions represent a set of six multiple choice questions, one per exercise, constructed in French by the scientific director of COVIRTUA healthcare, who had a good understanding of the exercises and whose native language is French.

## 3 Methods

**FIGURE 3:**
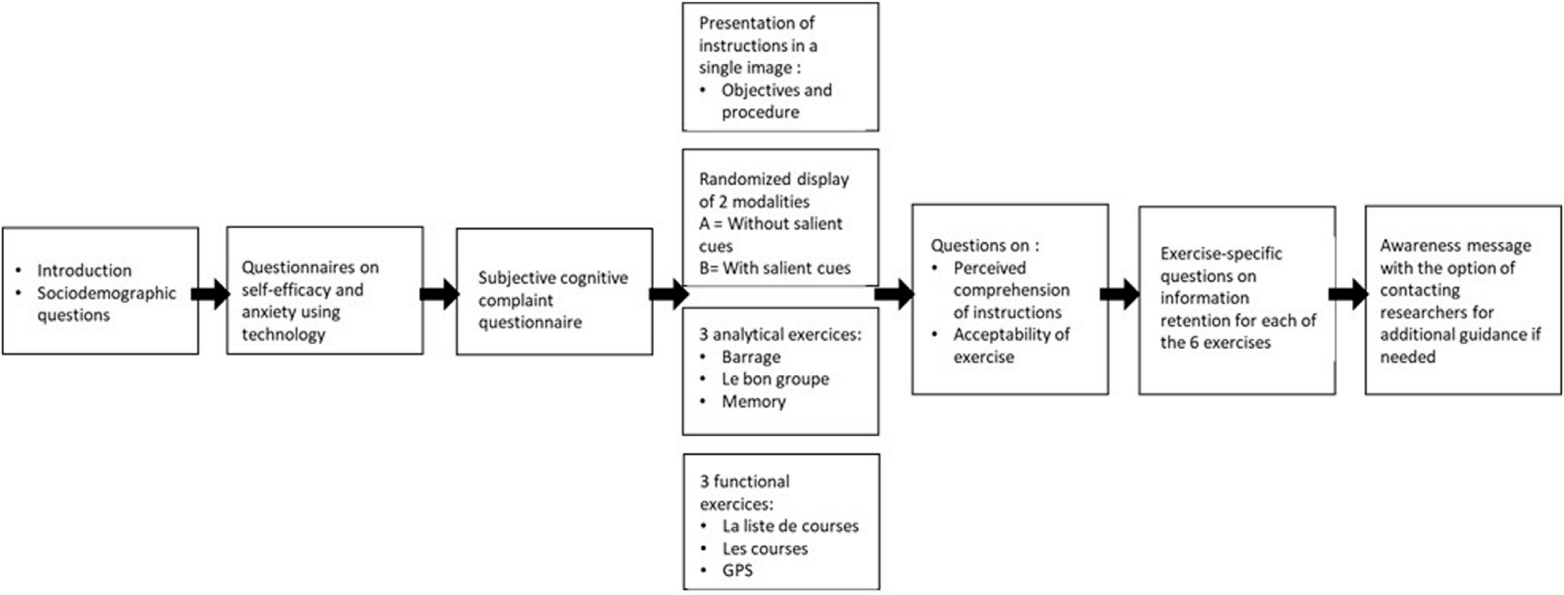
Steps of the assessment procedure.

This method adheres to ethical guidelines for research involving human subjects, which included obtaining approval from the university’s ethical board (CERGA-Avis-2023–18) and complying with European data rules as well as the Declaration of Helsinki. Figure 3 provides a detailed representation of the study’s procedure (Figure 3). The evaluation protocol will take approximately 25 min to complete, and was pre-tested by individuals over the age of 65 to insure its applicability.

**FIGURE 4:**
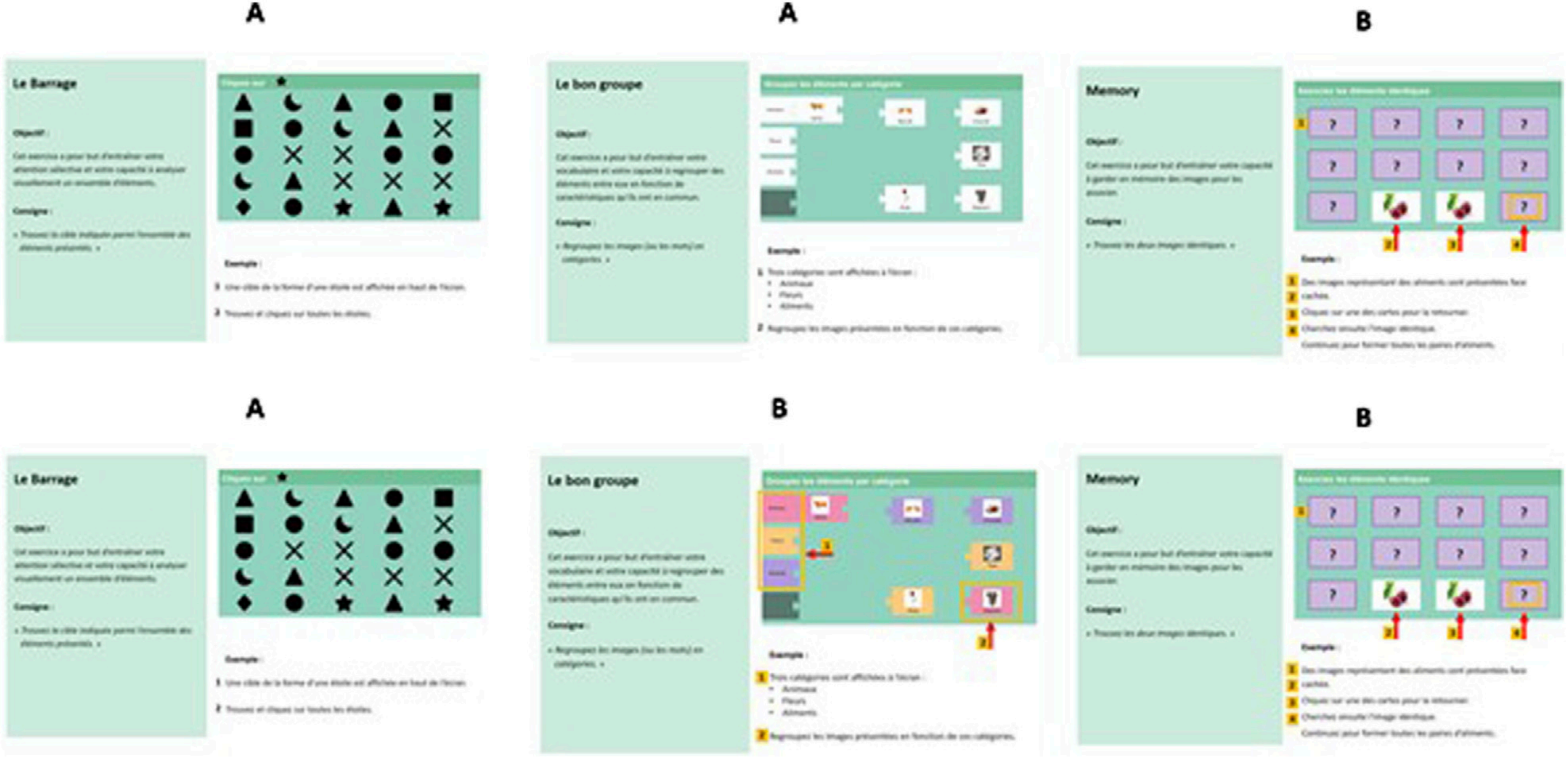
Example of possible counterbalanced orders for the presentation of the instructional material of the CCT serious games (AAB as seen above; ABB as seen below).

Step 1: Collection of informed consent after reading the informed information leaflet, socio-demographic questionnaire.
Step 2: Assessment of factors likely to influence acceptability and commitment to a technology (cognitive self-efficacy, technological self-efficacy and technology anxiety).
Step 3: Presentation of instructions for six CCT serious games (three analytical games and three functional games). Each game’s instructions will be presented in two blocks: Modality A (without visual cues) and Modality B (with visual cues such as arrows, colors, and shapes). The sequence of instruction presentation will be counterbalanced to eliminate order bias and carry-over effects, with participants experiencing one of six possible conditions for each block (AAB, ABB, BBA, BAA, ABA, BAB), presented randomly. A figure explaining two different presentation blocks is found below (Figure 4). Table 2 outlines the various blocks participants may encounter through this counterbalanced method. Although all participants will experience both instruction modalities, the specific sequence will differ according to the assigned blocks.
Step 4: Questions to assess understanding of the instructions and acceptability of each game following presentation of the instruction. These questions are derived from the Reduced Instructional Materials Motivation Survey (RIMMS) and the eHealth acceptability scale, assessing perceived need, outcome expectations, self-efficacy, and intention to use the CCT software.
Step 5: Specific questions to assess their retention of information from the instructions. The entire procedure, including the completion of questionnaires and instructional evaluations, is designed to take approximately 25 min. An awareness message will be displayed at the end of the questionnaire, offering participants the option to contact the research team for further guidance if desired. This study aims to enhance engagement in cognitive training and reduce dropout rates among older adults by identifying factors that influence the acceptance and comprehension of instructional designs.

**TABLE 2:** List of possible counterbalanced order for the presentation of the instructional material of the CCT serious games (Modality A = Without salient visual cues; Modality B = With salient visual cues).

Block 1 - analytical exercises	Le Barrage	Le Bon groupe	Memory
Order_1	A	A	B
Order_2	A	B	B
Order_3	B	B	A
Order_4	B	A	A
Order_5	A	B	A
Order_6	B	A	B

Block 2 - Functional exercises	GPS	Les courses	Liste de courses
Order_1	A	A	B
Order_2	A	B	B
Order_3	B	B	A
Order_4	B	A	A
Order_5	A	B	A
Order_6	B	A	B

## 4 Data analysis

To meet the study’s objectives and analyze the data consistently with the hypotheses, we selected statistical analyses suited to the comparisons and relationships examined, considering data distributions and measurement scales, in line with methodological recommendations (Conover et al., 1999; Gravetter et al., 2017). The paired comparisons between the within conditions will be done with independent samples *t*-test for normally distributed variables and Mann-Whitney *U*-test for non-normally distributed variables. Additionally, correlational tests (Pearson’s and Spearman’s rho correlation tests, respectively for normally distributed variables and non-normally distributed variables) will also be conducted a to examine the relationships between participants’ self-efficacy and feelings of anxiety related to technology, and their acceptance and understanding of the instructions presented with or without salient visual cues in the context of the CCT software. Finally, for control purposes we will perform a correlation analysis between SCCQ scores and comprehension/acceptability scores. This analysis will employ either Pearson’s correlation test or Spearman’s rank correlation test. Its purpose is to provide valuable insights into whether a subjective perception of cognitive capacities could potentially have a negative impact on the overall experience. Statistical data processing will be performed using Jamovi v.2.3.28. *P*-value ≤0.05 will be considered statistically significant.

## 5 Anticipated results

The anticipated results of our study focus on the hypothesized impact of salient visual cues on the comprehension and acceptability of CCT instructions among older adults. We expect that instructions incorporating salient visual cues, such as arrows, colors, and shapes, will significantly enhance both comprehension and acceptability compared to instructions without these cues. Visual aids are anticipated to direct attention to crucial elements, reduce cognitive load, and improve understanding and engagement. Furthermore, we predict a positive relationship between higher self-efficacy in cognitive abilities and technology use with better comprehension and acceptance, while higher levels of technology-related anxiety are expected to correlate negatively with these outcomes. We also foresee that better comprehension of instructions will lead to higher perceived usefulness and greater intention to use the CCT software.

However, potential limitations must be considered. The repeated nature of acceptability questions across six different games required us to limit the length of the questionnaire, possibly affecting the sensitivity of our measures. Nevertheless, to make it more accessible and reduce questionnaire time, we selected key elements central to acceptability, particularly those most strongly related to the construct (Boateng et al., 2018) instead of using long acceptability questionnaires. In addition, previous research has shown that intrapsychic variables such as self-efficacy and anxiety might not have a direct impact on the acceptability of training exercises or different modes of instruction presentation. This could be due to cognitive biases affecting acceptability scores. Studies by Ciriello et al. (2023) highlighted how cognitive biases like acquiescence and confirmation bias can create a paradox where high acceptability scores do not correspond to actual usage, while users with numerous complaints are more engaged (Ciriello et al., 2023). Furthermore, Righi et al. (2009) observed that highly anxious individuals often employ cognitive strategies to perform well, indicating a complex interaction between anxiety levels and cognitive self-assessment (Righi et al., 2009).

## 6 Discussion

There is a tight relationship between acceptability and usability, both of which affect user engagement (Lequerica et al., 2010; Alexandre et al., 2018; Nadal et al., 2020; Venkatesh et al., 2012; Davis et al., 1986; Bandura, 1982). The usability challenges faced by older adults have been documented in the literature (Nurgalieva et al., 2019; Barnard et al., 2013; Vroman et al., 2015), with lower computer proficiency and perceived ease of use being negatively linked to their long-term adoption of technology (Mitzner et al., 2019; Wildenbos et al., 2018; Wildenbos et al., 2019). Additionally, lower cognitive skills, specifically executive function skills, computer self-efficacy and technology anxiety have also been identified as challenged for long-term adoption of technology by this target population (Mitzner et al., 2019; Wildenbos et al., 2018). Even for individuals without a neurocognitive disorder, age related cognitive difficulties might affect their use of technology. Information processing, vision, audition and memory are all areas impacted negatively by the ageing process. (Farage et al., 2012; Morey et al., 2019; Nurgalieva et al., 2019). In response to these challenges, various research proposes design guidelines to facilitate the creation of suitable tools for this demographic (Sharit et al., 2020; Farage et al., 2012; Nurgalieva et al., 2019; Azevedo et al., 2022).

Instructional design affects information processing, therefore, holds a significant influence over comprehension (Ganier, 2004; Ganier, 2013a; Ganier, 2013b; Ganier et al., 2000; Sohlberg et al., 2011). Comprehension is an important pilar for effective usability (Kabir et al., 2016) as well as acceptability (perceived ease of use, self-efficacy, outcome expectancy) (Lequerica et al., 2010; Alexandre et al., 2018; Nadal et al., 2020; Venkatesh et al., 2012). Focusing on instructional design, could address cognitive and perceptual barriers associated to technology use by older adults such as: capacity of using technology (e.g., navigation, learning); information processing; memory; language (e.g., choice of wording) and visual needs (e.g., font size, colors) (Farage et al., 2012; Morey et al., 2019; Nurgalieva et al., 2019).

While numerous design guidelines exist (Farage et al., 2012; Chowdhury et al., 2021; Mitzner et al., 2019; Carmien et al., 2014; Boot et al., 2020; Czaja and Sharit, 2012) there is an absence of recommendations specifically addressing visual aids for computerized cognitive training software. Therefore, research on this topic could yield valuable insights into the preferences and comprehension of instructions presented with and without visual aids. The models we propose incorporate several recommended practices, including the integration of multimedia elements such as images and text (Ganier, 2004; Ganier, 2013a; Chowdhury et al., 2021; Mayer and Moreno, 2005). Additionally, we have implemented the strategy of breaking down information into smaller, manageable steps (Sohlberg et al., 2011; Morey et al., 2019; Azevedo et al., 2022; Sohlberg et al., 2005). Specifically, for the instruction models featuring visual aids, we have highlighted important information using icons and colors to ensure its saliency (Wynn et al., 2020; Davis and Ohman, 2016).

In this context, our study aims to demonstrate that incorporating salient visual cues in CCT instructions will improve comprehension and acceptability among older adults. By addressing factors such as self-efficacy and technology anxiety, we hope to identify key elements that influence engagement with CCT tools. The within-subject design of our study, which controls for individual differences, and the online methodology, which allows for broader participant reach and ensures compliance with data protection regulations, aims to provide robust insights that can inform the development of more effective cognitive training programs. Ultimately, we aim to enhance engagement and reduce dropout rates among older adults in cognitive training programs.

The choice of a within-subject design for the experiment provides benefits such as eliminating the need for random assignment, because each participant acts as their own control and experiences multiple conditions (modality A and B). It also allows for a smaller sample requirement while still maintaining strong internal validity and high statistical power (Charness et al., 2012). As an example, (Haggas et al., 2002), investigated student preference for overt vs covert responding in a web-based tutorial using a within-subject design. Only twenty-six social psychology students were exposed to the same two treatment conditions: covert question format (which required passive responding - “thinking” about an answer) and overt question format (which required active responding - “clicking” on an answer). A more recent study by (65) explored the effects of adding representational pictures to multiple-choice and constructed-response test items to understand the role of the response format for the multimedia effect in testing. They used a 2 × 2 within-subject design, two independent factors, multimedia (text-picture [TP] vs text-only [TO]) and response format (multiple-choice [MC] vs constructed-response [CR]) were varied in this study. The general design included four conditions (MC-TP, CR-TP, MC-TO, CR-TP), which were placed in a unique test booklet for each participant, so that each student completed test items in all four experimental conditions. Both studies purposefully adopted a within-subject design with the common objective of subjecting all participants to identical conditions, in order to evaluate the impact of each factor. For Haggas and Hantula (2002) this choice was also motivated by the desire to enhance the internal validity of their results while achieving heightened statistical power. Furthermore, both studies implemented counterbalancing techniques to control for potential biases and uphold the internal validity of their findings.

Using a within-subject design has some limitations. One possible liability is the emergence of a “demand effect.” This occurs when participants interpret the intentions of the experimenter and subsequently modify their behavior, whether consciously or unconsciously (Rosenthal, 1976; White, 1977). Consequently, these adjustments can lead to misleading effects and potentially impact the validity of the findings (Charness et al., 2012). Another one is based on the concept of evaluability. In fact, when comparing different options directly, some features, can be easier to compare than others. When we are required to compare two options, individuals tend to give more weight to the characteristics that are easily evaluable. Within-subject designs involve exposure to multiple options by the same participant. In such designs, participants may utilize different criteria to determine their preferences between the instructional designs (Charness et al., 2012). Moreover, if the order of the questions is not varied or the questions are not carefully designed to avoid response trends, it becomes challenging to differentiate between carry-over bias and genuine changes in preferences. Carry-over bias can occur when participants’ responses in one set of instructions influence their perception in subsequent sets, potentially leading to confounding results. In such cases, it becomes more difficult to discern whether the observed variations in responses are due to the order effect or genuine shifts in preferences (Charness et al., 2012). Also, it may be important to reflect on whether the timing of presentation for these visual aids is crucial to minimize cognitive load and visual clutter (Davis and Ohman, 2016). In fact, the visual aids we propose in the instructional models are not presented gradually or in the form of animations. This limitation raises the concern that participants may experience cognitive load when visualizing the presented visual aids within the instruction.

In our study, we took into consideration the possible limitations by varying the question order in a counterbalanced manner to prevent participants from being influenced by previous questions. We can, later on examine correlations between the order of questions and the responses provided. This analysis will help us determine whether the questions were answered independently or if there is an influence from the order in which they were presented. Simply changing the order of the questions may not be sufficient to solve the problem, therefore, by counterbalancing the presentation of the instructional material, each participant will undergo all experimental conditions, but the order of these conditions is systematically varied to prevent the influence of sequence effects and increase the accuracy of the study’s findings. As explained above, each participant will be exposed to all serious games included in both Block one and Block 2, as well as both types of instructions (A and B) as shown in the figures for instructional design. However, they will experience one of six conditions for each block (AAB–ABB–BBA–BAA–ABA- BAB), presented randomly. Although this helps in minimizing order bias and carry over bias, there are still risks to encounter demand effect and differences in evaluability.

## 7 Conclusion

The comprehension of instructions could influence the acceptability of technology as well as its usability. This is because understanding instructions requires the engagement of cognitive functions like information processing, memory, and perceptual visual skills. When impacted by aging or decline, these cognitive skills are susceptible to impact factors such as perceived ease of use, and computer self-efficacy. Struggles associated to technology use, lead to poorer acceptability of technology and lack of engagement potentially acting as barriers to effective utilization. By suggesting the above-mentioned within-subject experimental design our objective is to investigate the potential impact of self-efficacy and technology anxiety on instruction comprehension and acceptability in regards to the presence or absence of salient visual cues within CCT serious game instructions. By understanding what efficient ways there are to present instructions within this context, we would be closer to exploring potential facilitators to CCT use and long-term engagement.

## Data Availability

The original contributions presented in the study are included in the article/Supplementary Material, further inquiries can be directed to the corresponding author.
