# Supplementary material for: Engagement in computerized cognitive training instructions by older people. A within-subject design to evaluate comprehension and acceptability of serious games instructions
Source: Front Aging. 2025 Feb 3;6:1297704. doi: 10.3389/fragi.2025.1297704 (PMC11830709; doi:10.3389/fragi.2025.1297704)
Supplement: Supplementary file 1 [file DataSheet1.docx]

**Instructional material used for the study on:**

**Evaluating the perception of the instructions required to engage in computer cognitive training by older people, a within-subject design**

Christelle Nahas, Univ. Grenoble Alpes, CNRS, TIMC, 38000 Grenoble, France

Marc Gandit, Univ. Grenoble Alpes, LIP/PC2S, 38000 Grenoble, France

?, Covirtua Healthacare, 31770 Colomiers, France

Emmanuel Monfort, Univ. Grenoble Alpes, CNRS, TIMC, 38000 Grenoble, France

**French instructions for Computerized Cognitive Training serious games :**

Without visual cues (Modality A) – First image / With visual cues (Modality B) – Second image

English translation below

**Outline:**

**Analytical exercises:**

Le Barrage

Le Bon Groupe (The right group)

Memory

**Functional exercises :**

GPS

Les courses (Grocery shopping)

Liste de courses (Grocery list)

**Le Barrage**

**
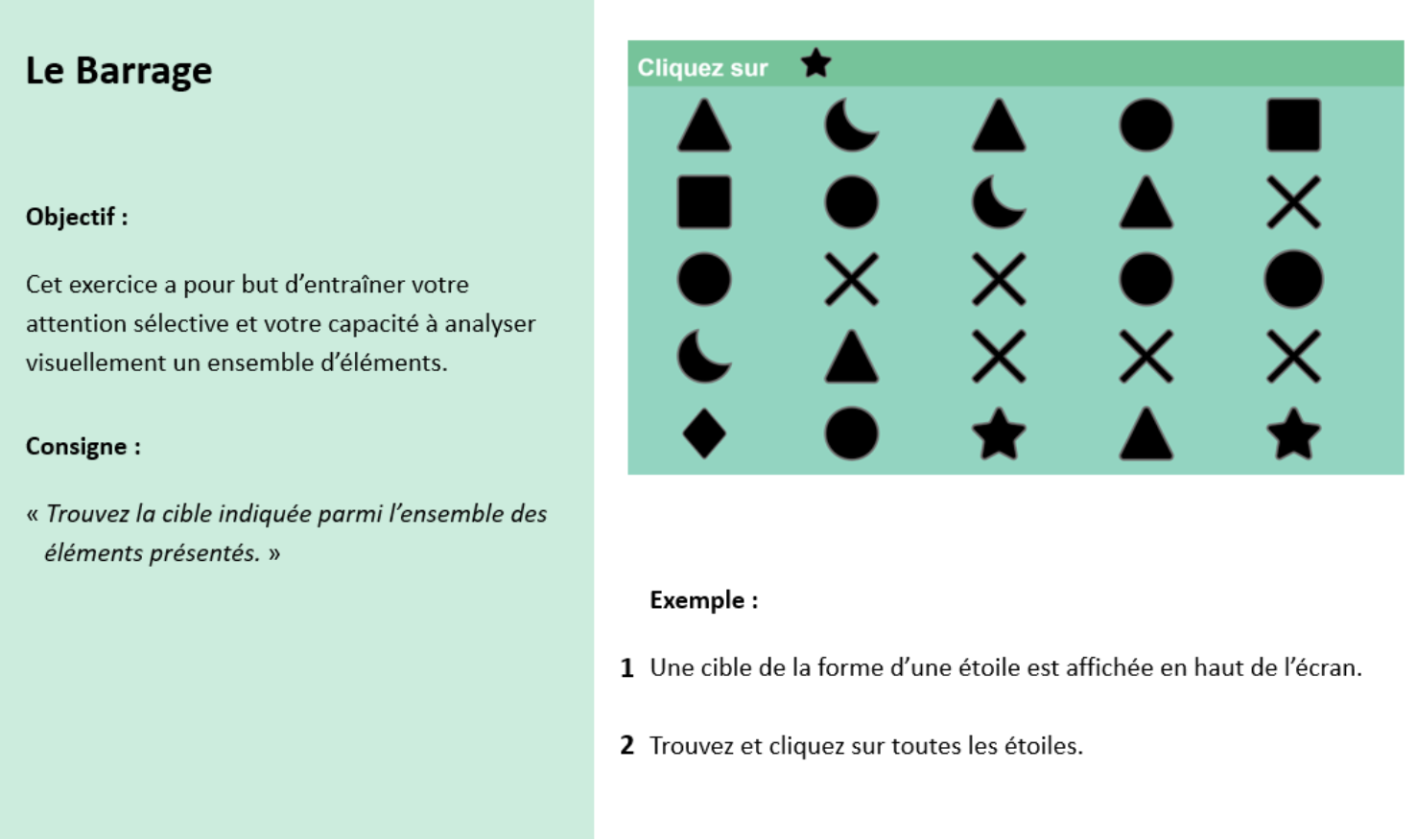
**


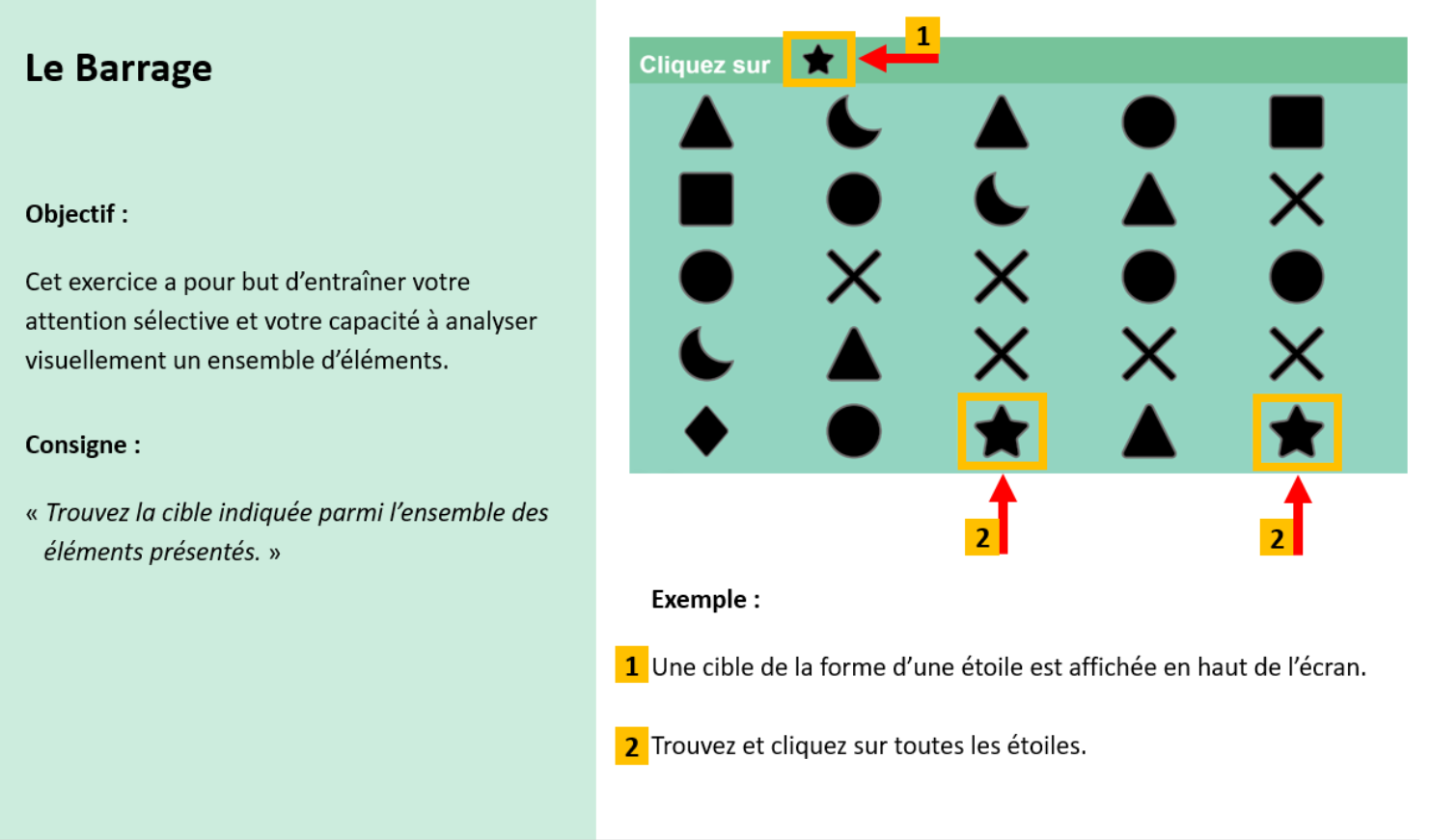


**Objective:** This exercise aims to train your selective attention and your ability to visually analyze a set of elements.

**Instruction:** “Find the indicated target among all the elements presented.”

**Example:**

1. **A star-shaped target is displayed at the top of the screen.​**
2. **Find and click on all the stars**

**Le Bon Groupe (The right group)**

**
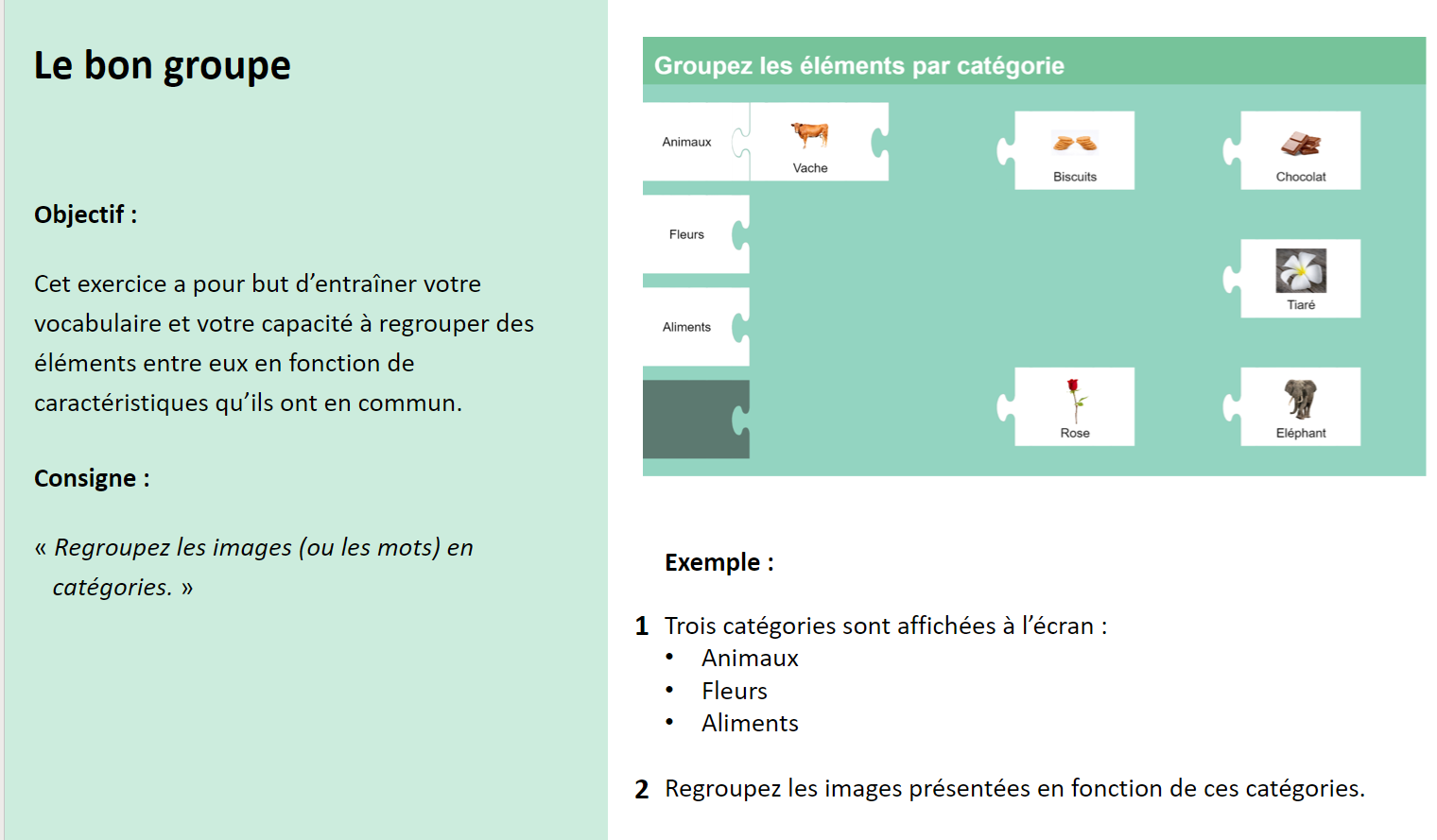
**

**
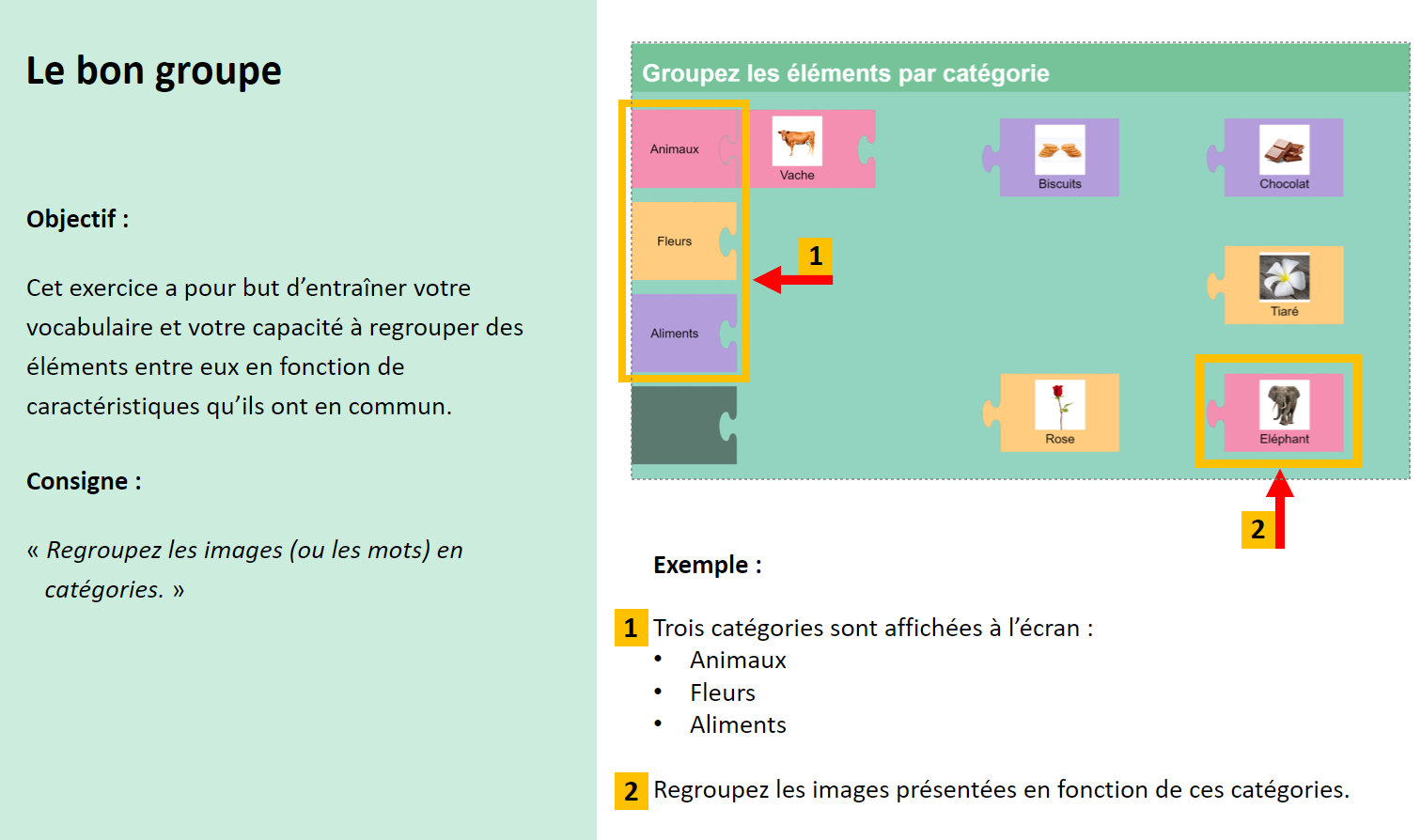
**

**Objective:** The purpose of this exercise is to train your vocabulary and your ability to group elements together according to the characteristics they have in common.

**Instruction:** “Group pictures (or words) into categories. »

**Example:**

1. Three categories are displayed on the screen: ​

- Animals​
- Flowers​
- Food​

1. Group the featured images according to these categories.

**Memory**

**
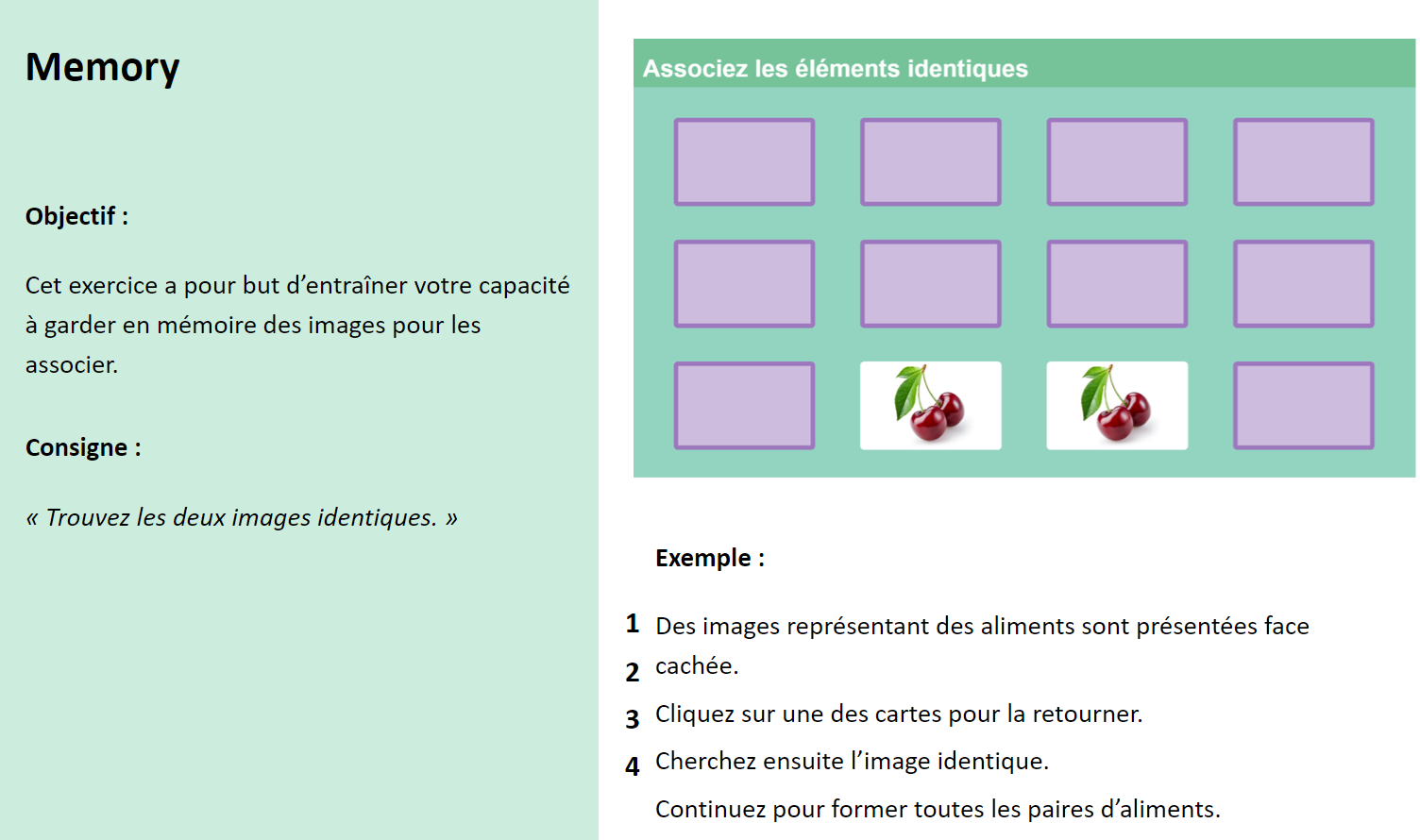
**

**
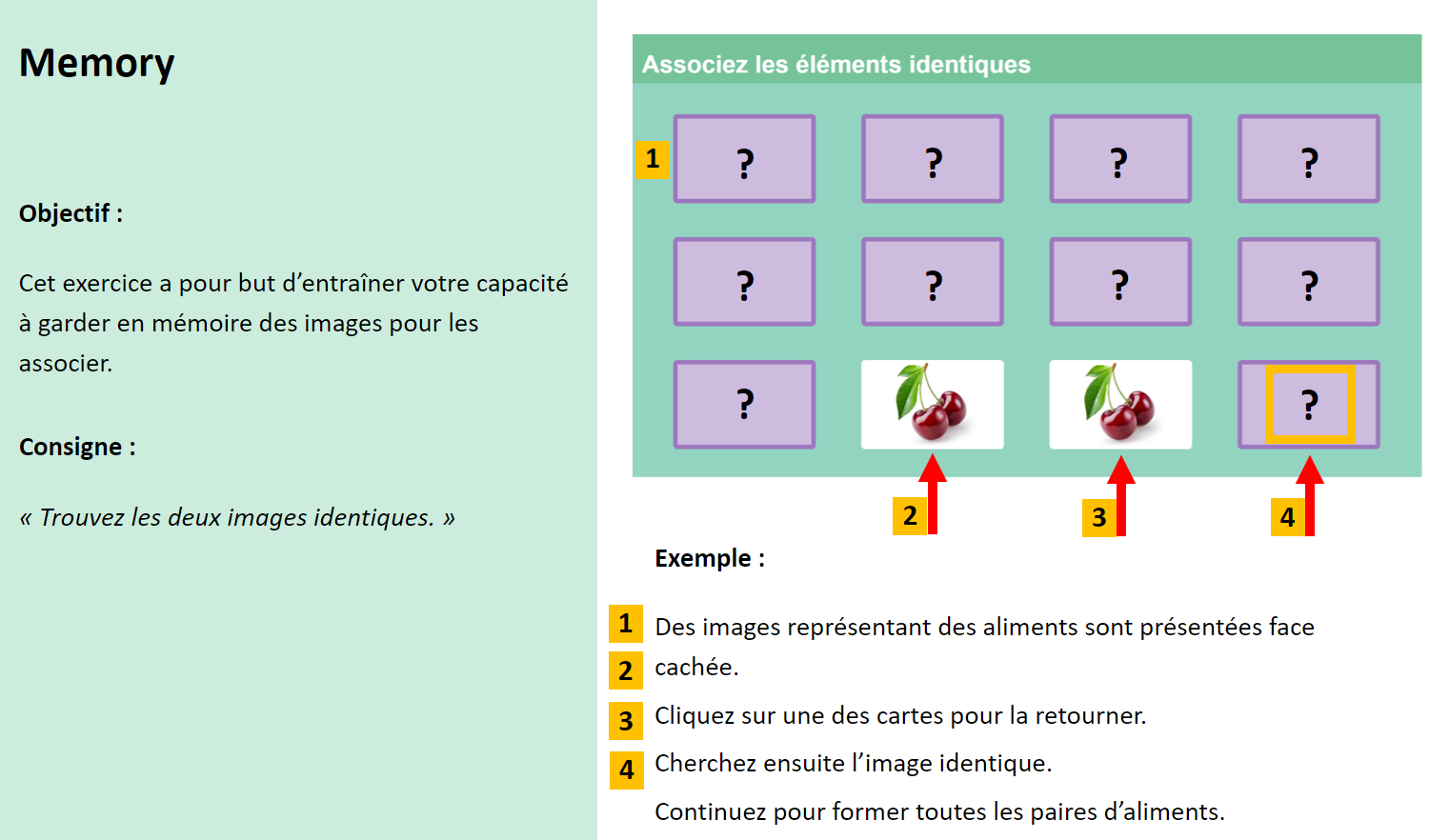
**

**Objective:** This exercise aims to train your ability to remember images in order to pair them

**Instruction:** "Find the two identical images.”

**Example:**

1. Pictures representing food are presented face down.​
2. Click on one of the cards to turn it over.​
3. Then look for the identical image.​
4. Continue to form all food pairs.

**GPS**

**
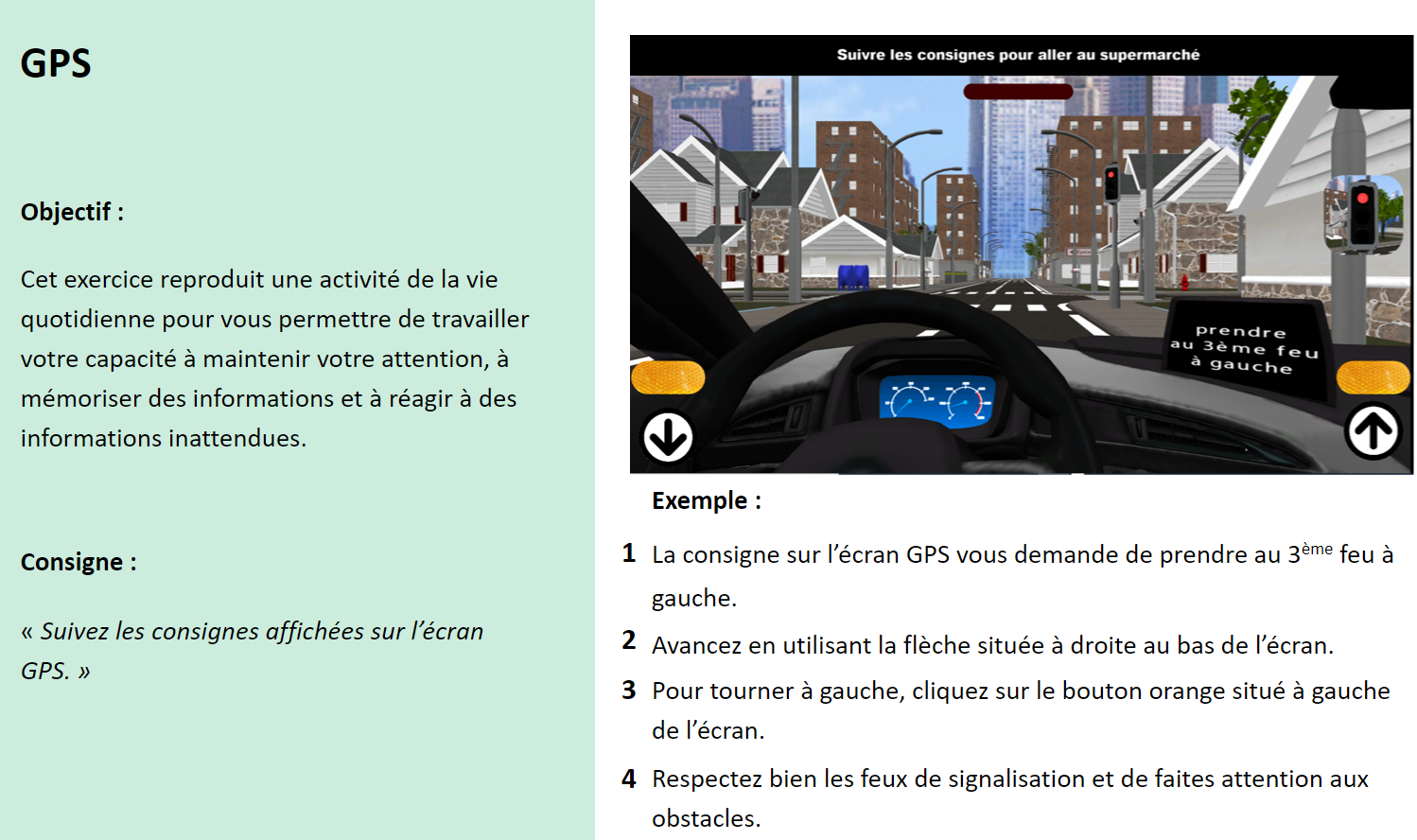
**

**
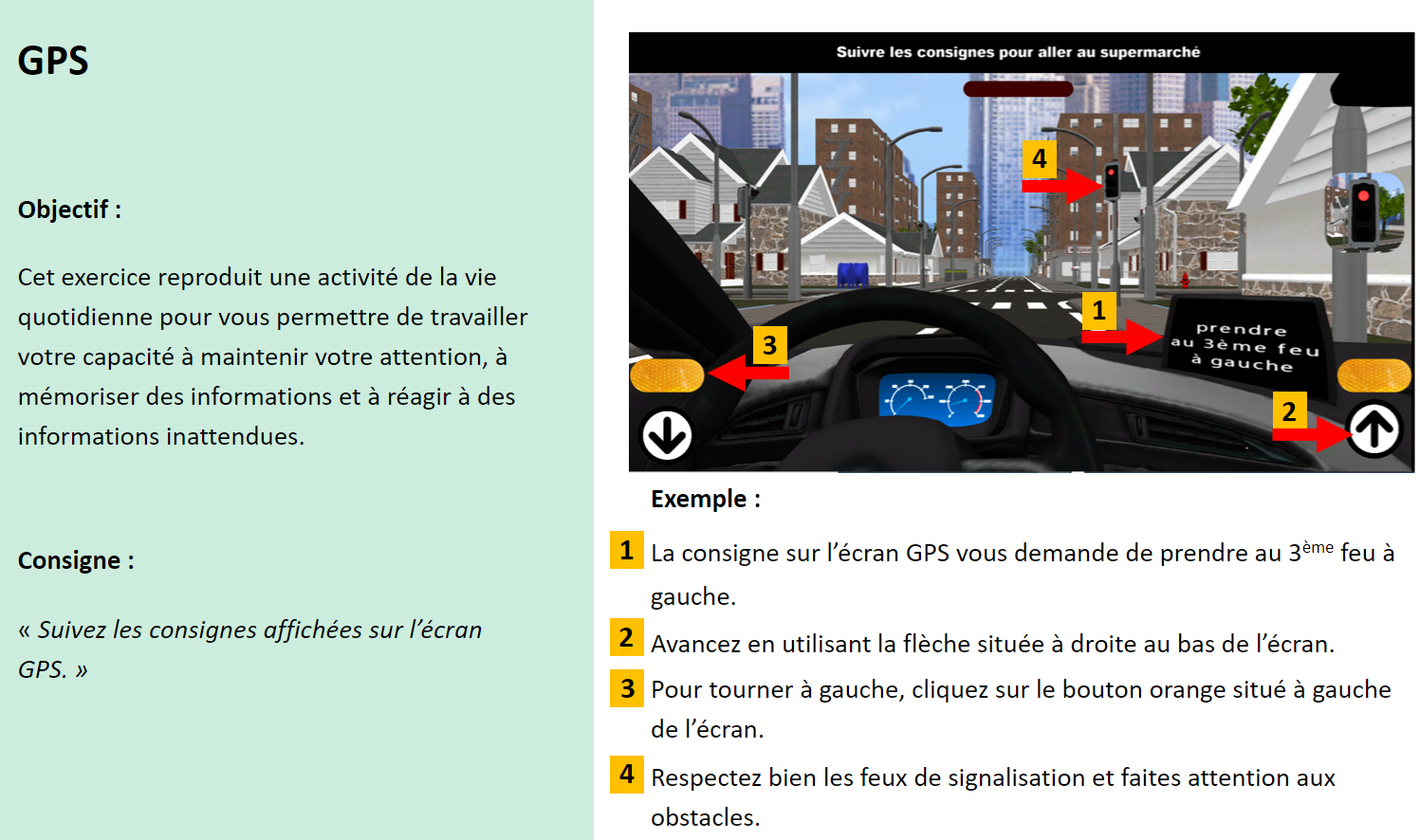
**

**Objective:** This exercise is a simulation of a daily life activity to work on your ability to maintain your attention, memorize information and react to unexpected information.

**Instruction:** “Follow the instructions displayed on the GPS screen.”

**Example:**

1. The instruction on the GPS screen asks you to take the 3rd traffic light on the left.​
2. Move forward using the arrow on the right at the bottom of the screen.
3. To turn left, click the orange button on the left side of the screen.​
4. Respect the traffic lights and watch out for obstacles.

**Les courses (Grocery shopping)**

**
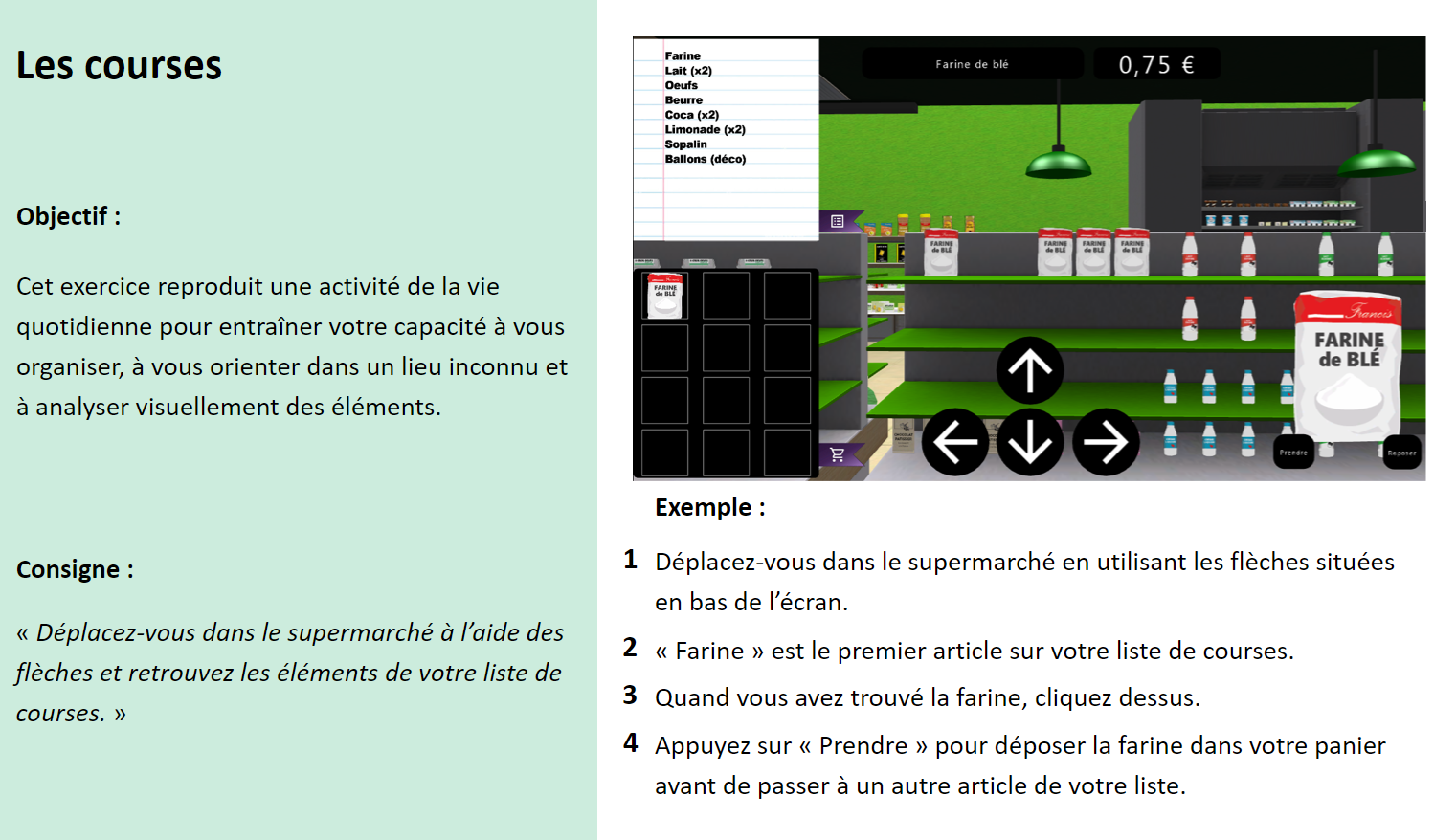
**

**
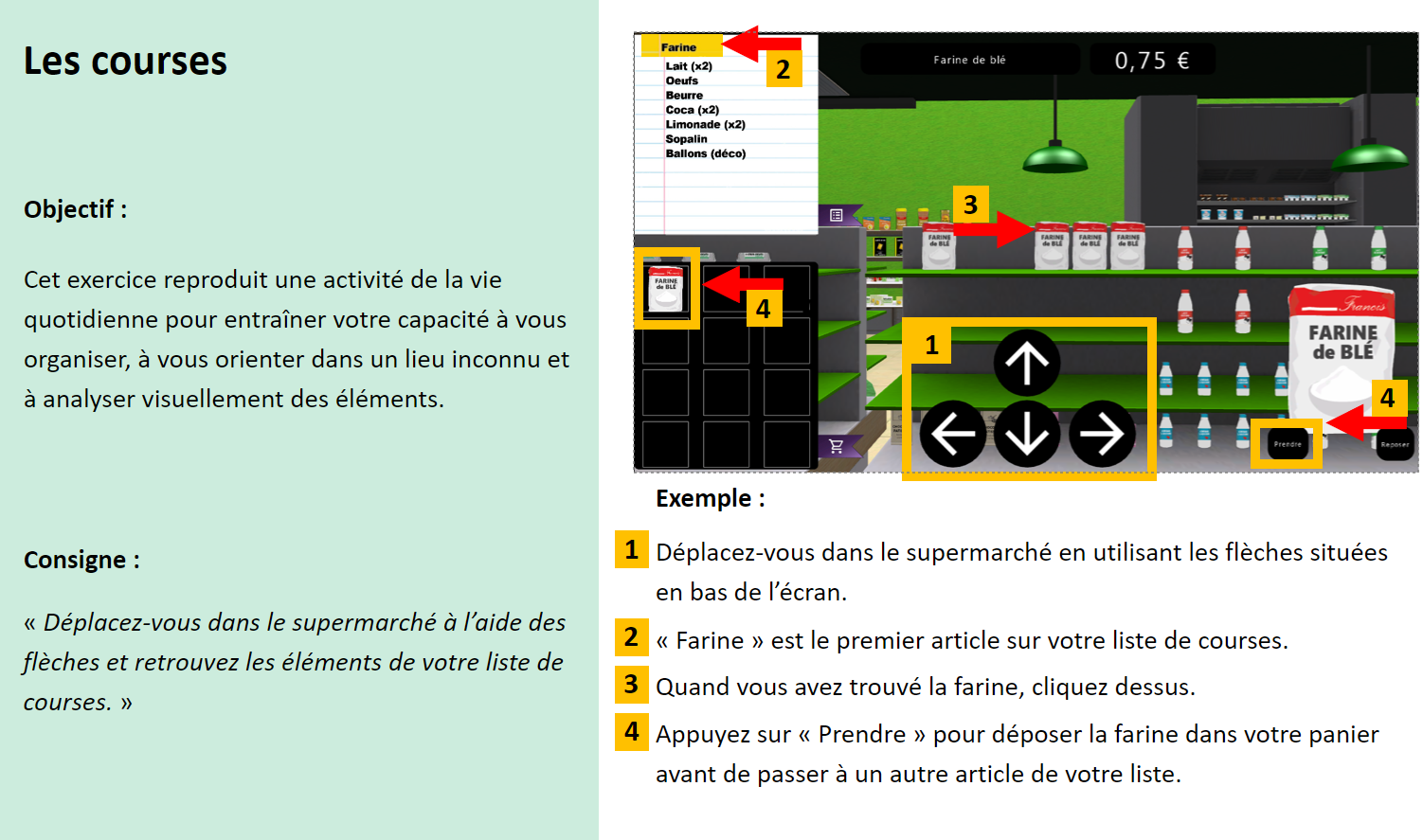
**

**Objective:** This exercise is a simulation of a daily life activity to train your ability to organize yourself, to orient yourself in an unfamiliar place and to visually analyze elements.

**Instruction:** “Move through the supermarket using the arrows and find the items on your shopping list.”

**Example:**

1. Move around the supermarket using the arrows at the bottom of the screen.​
2. “Flour” is the first item on your shopping list.
3. When you find the flour, click on it.​
4. Press "Take" to put the flour in your basket before moving on to another item on your list.

**Liste de courses​ (Grocery list)**

**
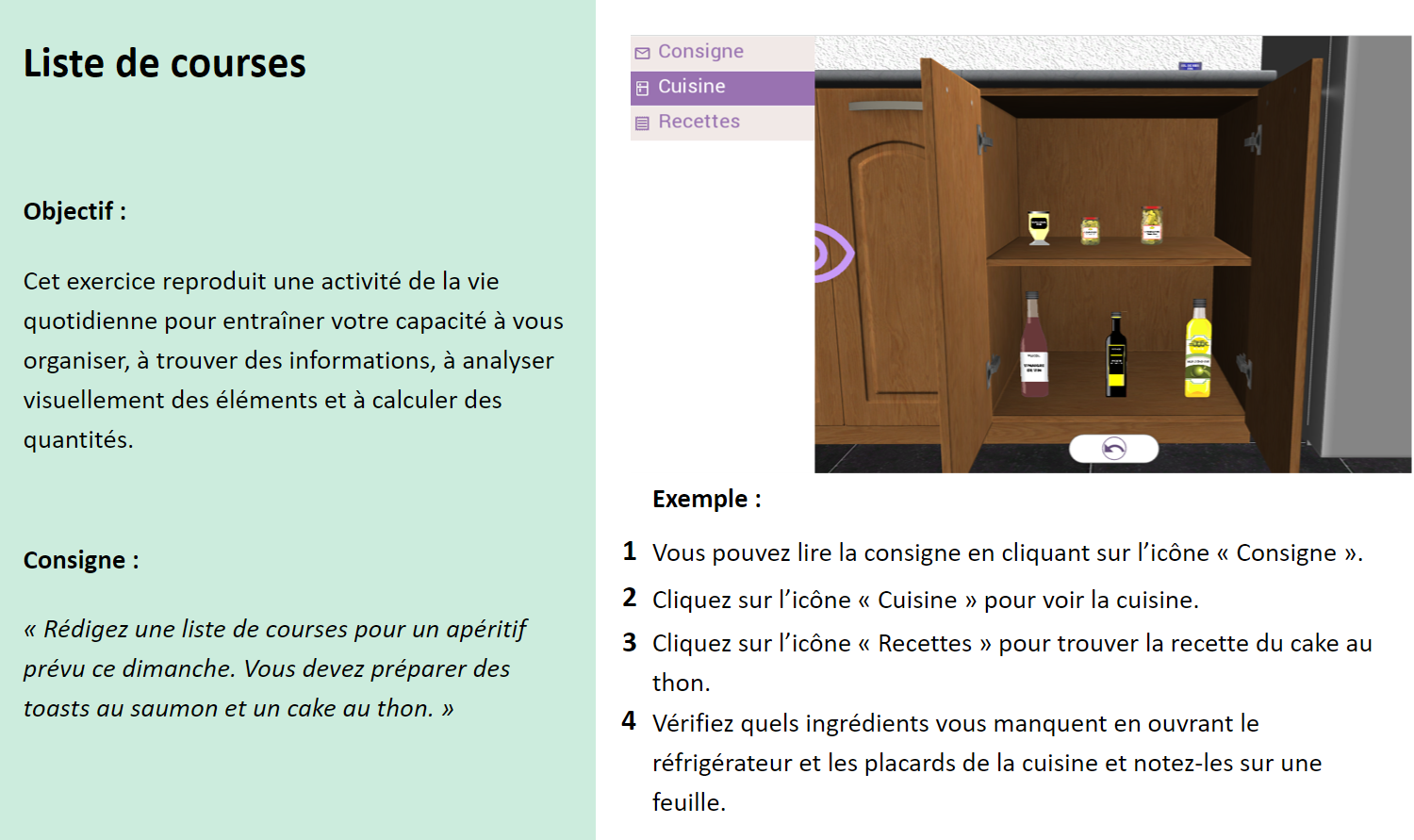
**

**
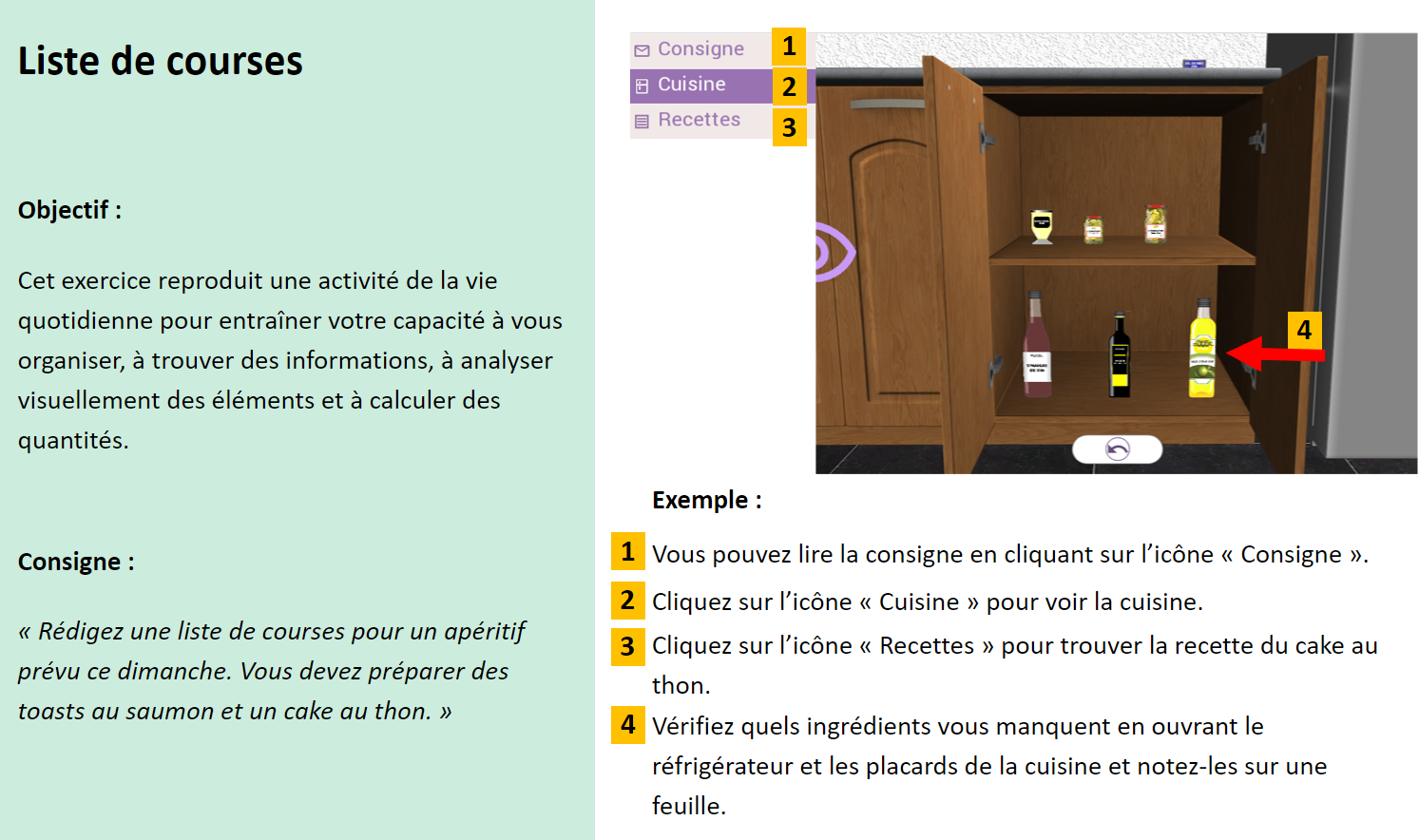
**

**Objective:** This exercise is a simulation of a daily life activity to train your ability to organize yourself, find information, visually analyze items and calculate quantities.​

**Instruction:** “Write a grocery list for an aperitif scheduled for this Sunday. You must prepare salmon toast and a tuna cake. »

**Example:**

1. You can read the instruction by clicking on the "Instruction" icon.
2. Click on the “Kitchen” icon to see the kitchen.
3. Click on the "Recipes" icon to find the recipe for tuna cake.​
4. Check what ingredients you are missing by opening the refrigerator and kitchen cupboards and write them down on a piece of paper.
